# HuMSC-EV induce monocyte/macrophage mobilization to orchestrate neovascularization in wound healing process following radiation injury

**DOI:** 10.1038/s41420-023-01335-y

**Published:** 2023-02-01

**Authors:** Céline Loinard, Alexandre Ribault, Bruno Lhomme, Marc Benderitter, Stéphane Flamant, Sandrine Paul, Valérie Dubois, Ruenn Chai Lai, Sai Kiang Lim, Radia Tamarat

**Affiliations:** 1 Céline Loinard, Alexandre Ribault, Bruno Lhomme, Marc Benderitter, Stéphane Flamant, and Radia Tamarat, Institut de Radioprotection et de Sûreté Nucléaire (IRSN), PSE-SANTE, Fontenay-aux-Roses, France; 2 Sandrine Paul & Valérie Dubois, EFS Auvergne Rhône Alpes, laboratoire HLA, 111, rue Elisée-Reclus, Lyon, 69150 Décines France; 3 Ruenn Chai Lai & Sai Kiang Lim, Institute of Medical Biology A*STAR, #05-16 Immunos, & Institute of Molecular and Cellular Biology, A*STAR, #05-38 Immunos, 8 A Biomedical Grove, Singapore, 138648 Singapore

**Keywords:** Mesenchymal stem cells, Mechanisms of disease

## Abstract

This study aims to investigate the mechanisms of human mesenchymal stem cell-derived extracellular vesicles (HuMSC-EV)-induced proangiogenic paracrine effects after radiation injury. HuMSC-EV were locally administered in mice hindlimb following 80-Gy X-ray irradiation and animals were monitored at different time points. HuMSC-EV improved neovascularization of the irradiated tissue, by stimulating angiogenesis, normalizing cutaneous blood perfusion, and increasing capillary density and production of proangiogenic factors. HuMSC-EV also stimulated vasculogenesis by promoting the recruitment and differentiation of bone marrow progenitors. Moreover, HuMSC-EV improved arteriogenesis by increasing the mobilization of monocytes from the spleen and the bone marrow and their recruitment into the muscle, with a pro-inflammatory potential. Importantly, monocyte depletion by clodronate treatment abolished the proangiogenic effect of HuMSC-EV. The critical role of Ly6C(hi) monocyte subset in HuMSC-EV-induced neovascularization process was further confirmed using *Ccr2*^*−/*−^ mice. This study demonstrates that HuMSC-derived EV enhances the neovascularization process in the irradiated tissue by increasing the production of proangiogenic factors, promoting the recruitment of vascular progenitor cells, and the mobilization of innate cells to the injured site. These results support the concept that HuMSC-EV might represent a suitable alternative to stem cells for therapeutic neovascularization in tissue repair.

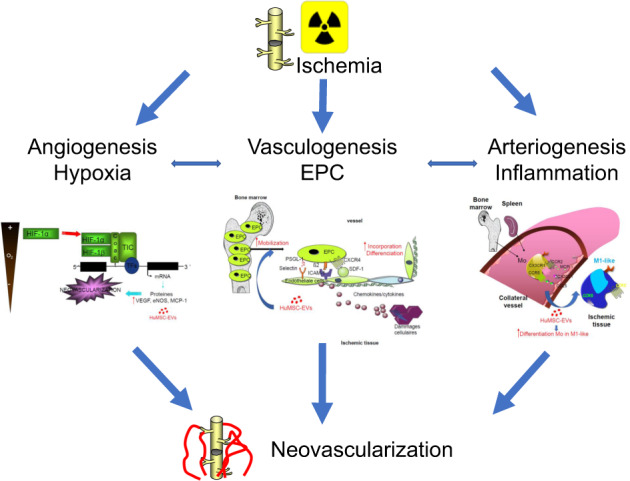

## FACTS

We were able to link that

1. HuMSC-EV injection improves neovascularization
  by stimulating angiogenesis
  by stimulating the vasculogenesis
  by improving arteriogenesis and increasing the mobilization of both Ly6C(hi) and Ly6C(lo) monocyte subsets from spleen and bone marrow into the muscle.
2. We further demonstrate the critical role of Ly6C(hi) monocytes in HuMSC-EV-induced neovascularization process, through clodronate-induced monocyte depletion and use of *Ccr2*^*−*^^*/−*^ mice.
3. Additionally, studies for optimizing the beneficial effect on neovascularization process and functional muscle regeneration will be necessary to determine the dose, the number, and route of injection on need it.

## Introduction

Significant radiation induced complication is a concern for 95% of patients receiving radiation therapy (RT) for cancer treatment at long term. In addition, ionizing radiation is also a public health concern due to the risk of a nuclear and/or radiological event. The management of such complications would improve the therapeutic benefit of cancer treatment by RT and the expected morbidity in any mass casualty “terrorist” attack.

One major complication of local radiation injury is deficient wound repair arising from inadequate reepithelization and neovascularization [1]. In fact, the process of neoangiogenesis occurs during adult wound healing and is essential for wound repair. The neoangiogenesis process involves the formation and the remodeling of new blood vessels from pre-existing ones. Vasculogenesis occurs through the mobilization of progenitor cells, and arteriogenesis by inflammatory cell recruitment. These processes involve the recruitment of spleen- and bone marrow (BM)-derived cells, and the subsequent incorporation of BM-derived cells in the newly forming vessels [2, 3]. Previous studies demonstrated that these principal events are altered after radiation exposure [4–6]. Multiple processes promoting neovascularization are under the control of the master regulator hypoxia-inducible transcription factor (HIF). In normoxia, HIF-1α is hydroxylated at 2 critical prolines by the prolyl hydroxylase domain proteins (PHD)-1, 2 and 3, leading to HIF-1α ubiquitylation and subsequent proteasome mediated degradation [7, 8]. Hypoxic conditions compromise PHDs activity, leading to stabilization and nuclear translocation of HIF-1α, where it dimerizes with its constitutive nuclear partner HIF-β, and binds to hypoxia-responsive elements of target genes, including endothelial nitric oxide synthase (eNOS), monocyte chemoattractant protein-1/chemokine (C-C motif) ligand-2 (MCP-1/CCL2), and vascular endothelial growth factor (VEGF) [9].

Different mechanisms underlying blood vessel growth alterations after irradiation have been suggested, including a reduction in VEGF-A signaling, and changes in inflammation-related pathways. In addition, reactive oxygen species (ROS) including superoxide, hydrogen peroxide, hydroxyl radical, as well as reactive nitrogen species, such as nitric oxide and peroxynitrite, are abundantly produced after irradiation, and involved in vascular biology through redox signaling [10]. In fact, an increase in oxidative stress affects angiogenesis, vasculogenesis and arteriogenesis [11].

We recently showed the key role for monocyte/macrophage activation in the neovascularization process in a mouse model of local colorectal irradiation [12]. These inflammatory cells, consisting of Ly6C(hi) and Ly6C(lo) monocytes (Mo^hi^ and Mo^lo^), have been shown to modulate tissue remodeling and function [13, 14]. The recruitment of monocytes to ischemic areas occurs mainly via chemokine/chemokine receptor signaling. In ischemic hindlimb models, deficiency in CCL2 or its receptor, chemokine (C–C motif) receptor 2 (CCR2), reduces post-ischemic inflammatory reaction and neovascularization [10]. Interestingly, the absence of CCR2 specifically abolishes Mo^hi^ infiltration in the ischemic myocardium [15].

Recent studies have demonstrated that extracellular vesicles (EV) produced by BM-derived MSC can promote angiogenesis in several pathological conditions, including cutaneous wound healing, acute kidney injury and myocardial infarction, thereby contributing to tissue repair [16–18]. Accumulating evidences suggest that the beneficial effect of MSC is achieved mainly via paracrine mechanisms, characterized by the release of cytokines, growth factors, chemokines and more recently EV [19, 20]. EV are lipid bilayer membrane-bound vesicles released by virtually all cell types, which contain a variety of functional proteins, lipids and RNAs [21]. EV constitutes important cell-to-cell communication mediators in physiological and pathological conditions [22].

In the present study, we investigated the therapeutic and proangiogenic, provasculogenic and proarteriogenic effects of human MSC-derived EV (HuMSC-EV) in a mouse model of radiation injury. We analyzed their effect on the production of proangiogenic factors, as well as on the mobilization and recruitment of vascular progenitors and innate immune cells, and their role in the neovascularization process.

## Results

### HuMSC-EV ameliorate injury score and induce neovascularization

**Fig. 1:**
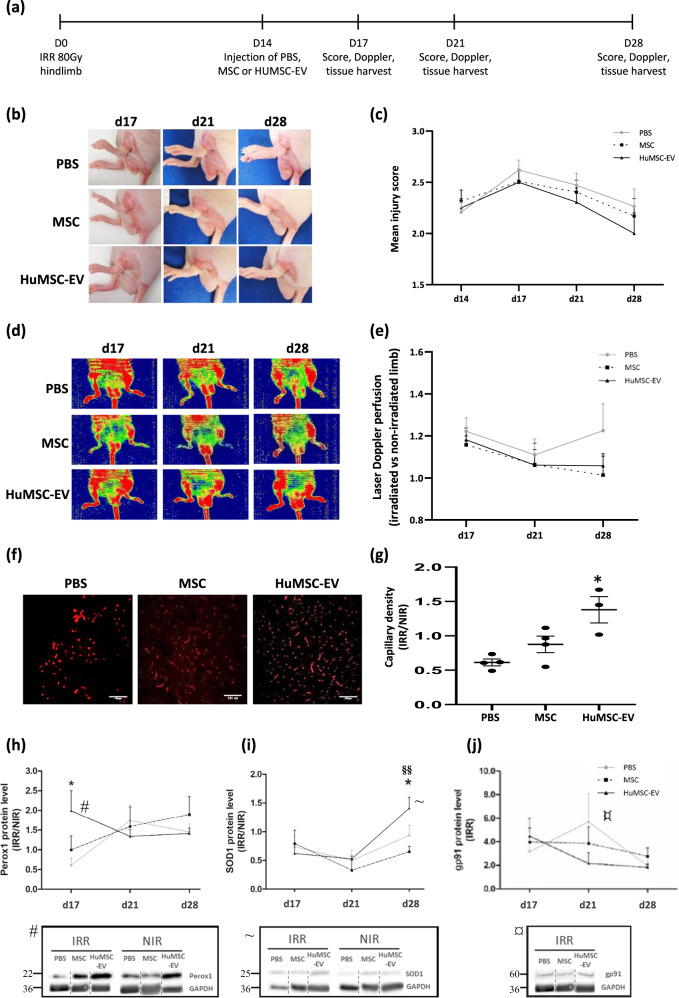
HuMSC-EV improve injury score and induce neovascularization in irradiated tissue. **a** Timeline of the experimental procedure. HuMSC-EV, MSC or PBS was injected at d14 after irradiation, and mice were analyzed 3, 7 and 14 post-injection (ie d17, 21 and 28 post-irradiation). **b** Representative photographs of radiation-induced hind limb injuries at different time points following injection of PBS (top), MSC (middle) or HUMSC-EV (bottom). **c** Kinetic evolution of injury scores following injection of PBS (circles), MSC (squares) or HuMSC-EV (triangles). *N* = 18–36 animals per time point (three independent experiments). **d**, **e** Laser Doppler blood flow analysis of animals in irradiated (right) and non-irradiated (left) limbs at days 17, 21, and 28 post-irradiation. **d** Representative images and **e** quantification of cutaneous perfusion in all groups. Results are expressed as the ratio of skin perfusion (arbitrary units) in irradiated limb to that of the non-irradiated limb. Stitch line represents the ratio for non-irradiated animals. *N* = 5–16 animals per time point. **f**, **g** Immunohistological analysis of capillaries in muscle tissue. **f** Representative pictures of CD31-labeled intramuscular blood vessels in irradiated muscle, injected with PBS (left), MSC (middle) and HUMSC-EV (right), 10 days after injection (d24 post-irradiation). **g** Ratio of capillary density in the irradiated limb to the capillary density of non-irradiated limb. Tissue protein expression levels of NADPH oxidase subunit Perox1 (**h**), SOD1 (**i**) and gp91-*phox* (**j**), in each group of mice. Symbols make the link between the highest fold difference during the time course analysis and the representative Western blots (d17 for Perox1, d28 for SOD1 and d21 for gp91-phox). Data in are medians ± SEM (*n* = 5 per group). Data in (i) and (j) are normalized to the corresponding protein level in non-irradiated muscle; *P < 0.05, compared to PBS; §§*P* < 0.01, compared to MSC; one-way ANOVA followed by Tukey’s test.

To investigate the therapeutic effect of HuMSC-EV we first assessed the kinetic of evolution of the lesion by a semi quantitative analysis of the wound extent, ulceration, moist desquamation and limb retraction during the wound healing process at 3, 7, and 14 days after injection (i.e., 17, 21, and 28 days post-irradiation, Fig. 1a). At day (D) 28 post irradiation, we observed that injection of HuMSC-EV and MSC tended to decrease the injury score compared to PBS-injected animals (Fig. 1b, c). More interestingly, HuMSC-EV and MSC tended to prevent the negative evolution of injury score observed at D17 compared to PBS-injected animals.

Skin blood perfusion of irradiated and non-irradiated limbs was measured by laser Doppler imaging (Fig. 1d, e). First, our results showed that irradiation induced an increase in skin perfusion by approx. 1.2-fold compared to the NIR animals at D17, in all groups (*P* < 0.001; Fig. 1e). Secondly, both HuMSC-EV and MSC injection restored the foot blood flow ratio to a level similar to non-irradiated condition at D28.

Changes in skin perfusion were associated with modification of capillary density. At D17, HuMSC-EV administration led to a significant increase of capillary density by 2.18-fold compared to PBS control animals (*P* < 0.05; Fig. 1f, g).

Interestingly, Western-blot analysis showed that HuMSC-EV induced a significant increase in tissue expression of Perox1 by 3.3-fold at D17 (*P* < 0.05) and SOD1 by 1.5-fold (*P* < 0.01) at D28, compared to PBS-injected animals (Fig. 1h, i and Supplemental Data Online). However, no modulation of the protein expression level of NADPH oxidase subunit gp91-*phox* was observed (Fig. 1j and Supplemental Data Online). Our results demonstrate that HuMSC-EV administration induced the production of antioxidant factors that act to suppress the formation of free radicals or reactive species in irradiated tissues.

### HuMSC-EV increase vasculogenesis and angiogenesis

**Fig. 2:**
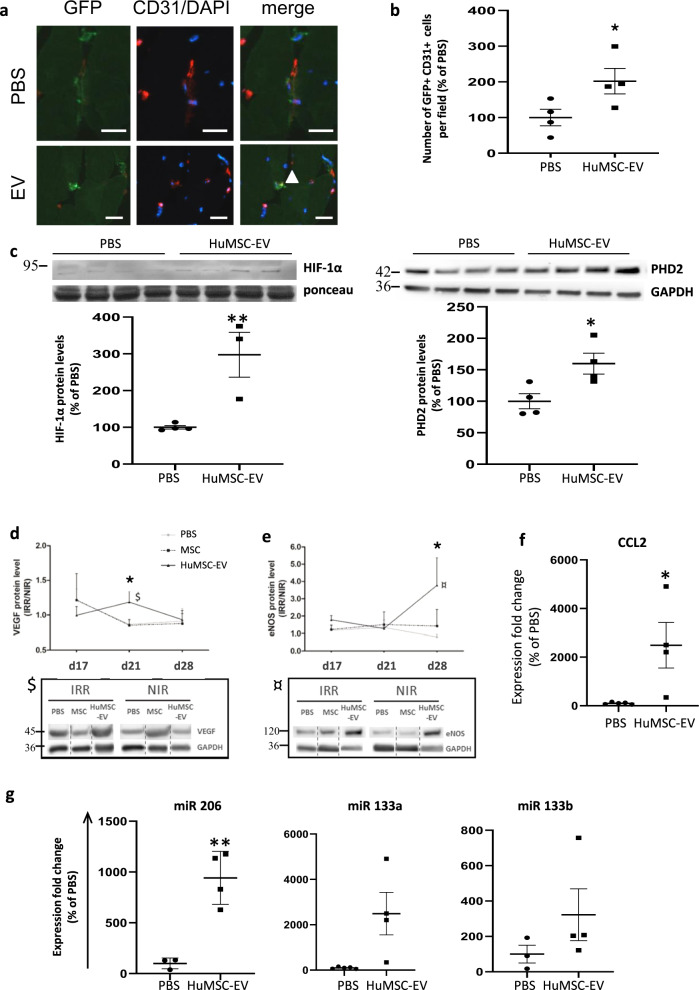
HuMSC-EV promote vasculogenesis, angiogenesis, and expression of skeletal muscle pro-regenerative miRNA. **a** Representative photomicrographs of incorporated BM-derived GFP+-CD31+ cells (GFP-positive cells appear in green, CD31 positive cells in red; nuclei were stained with 4’,6-diamidino-2-phenylindole [blue]) in chimeric mice injected with PBS or HuMSC-EV. Bar: 25 µm. **b** Quantitative evaluation of GFP + cells in irradiated muscle in chimeric mice injected with PBS or HuMSC-EV, 3 days after injection. Data are means ± SEM (*n* = 4 animals/group; 5–8 independent fields/animal, *P < 0.05, two-tailed Mann–Whitney). **c** Representative Western blot and quantification of HIF-1α and PHD2 protein levels in animal groups injected with PBS or HuMSC-EV, 3 days after injection. Protein level of angiogenic factors VEGF (**d**) and eNOS (**e**) in muscle tissue were assessed by Western-blot. Symbols make the link between the highest fold difference during the time course analysis and the representative Western blots (*P < 0.05 between HuMSC-EV- and PBS-injected groups, twp-tailed Mann–Whitney). **f** mRNA expression of proarteriogenic factor CCL2 in muscle tissue was assessed by qRT-PCR in PBS and HuMSC-EV-injected groups 3 days after injection. **g** Expression levels of skeletal muscle pro-regenerative miRNA-206, -133a and 133b were assessed by qRT-PCR in PBS- and HuMSC-EV-injected groups 3 days after injection. Data are means ± SEM (*n* = 5–6 animals/group). Data in (**d**) and (**e**) are normalized to the corresponding protein level in non-irradiated muscle; *P < 0.05; **P < 0.01; two-tailed Mann–Whitney).

We investigated the effect of HuMSC-EV on endothelial vascular progenitor cell differentiation in irradiated tissue. In order to evaluate the recruitment of BM-derived cells in the tissue, we generated chimeric mice by transplantation with BM isolated from GFP^+^ animals. We showed that HuMSC-EV significantly increased the number of GFP^+^CD31^+^ cells in the irradiated tissue compared with PBS-injected animals (*P* < 0.05; Fig. 2a, b), suggesting a vasculogenic effect of HuMSC-EV.

We next sought to determine the molecular and cellular pathways stimulating the neovascularization by HuMSC-EV in radiation injury. We analyzed HIF-1α and PHD1, 2 and 3 proteins in irradiated gastrocnemius, as well as HIF-dependant proangiogenic factors VEGF and eNOS, and the proarteriogenic molecule CCL2. These proteins are involved in stem cell-mediated vessel growth by action on local cell survival and cell mobilization, respectively. HIF-1α protein expression was increased by 3-fold in the HuMSC-EV-injected muscles at D17, compared to PBS-injected animals (*P* < 0.01; Fig. 2c and Supplemental Data Online). PHD1 and PHD3 expression levels were similar between the 2 groups (see Supplementary Fig. S1 and Supplemental Data Online). However, PHD2 expression was significantly increased by 1.5-fold in the irradiated muscle after HuMSC-EV administration compared to PBS-injected animals (*P* < 0.05; Fig. 2c & supplemental data online). Compared to PBS, HuMSC-EV administration resulted in significantly increased levels of tissular VEGF protein at D21 (1.4-fold, *P* < 0.05; Fig. 2d and Supplemental Data Online), eNOS protein at D28 (4.7-fold, *P* < 0.05; Fig. 2e and Supplemental Data Online), as well as CCL2 mRNA expression at D17 (24-fold, *P* < 0.05; Fig. 2f). These results demonstrated that HuMSC-EV induced the production of proangiogenic, proarteriogenic and prosurvival growth factors in irradiated tissue.

### HuMSC-EV promote muscle pro-regenerative microRNA expression

In addition, we evaluated the muscle response to HuMSC-EV injection by measuring the expression of skeletal muscle pro-regenerative microRNAs (miRNAs). Interestingly, the expression of miR-206 and miR-133 families increased by 9- and 3-fold, respectively, in the irradiated muscle injected with HuMSC-EV compared to PBS-injected animals at D17 (*P* < 0.05; Fig. 2g).

### HuMSC-EV stimulate wound healing process in an in vitro scratch model

To investigate the molecular mechanisms of action of HuMSC-EV, we analyzed their effect on the wound healing process. HuMSC-EV enhanced the migration of irradiated human dermal endothelial cells and dermal fibroblasts (see Supplementary Fig. S2 online), as compared to PBS control. Interestingly, treatment with the PI3K/AKT inhibitor LY294002 significantly inhibited HuMSC-EV-mediated effect on both endothelial cells (*P* < 0.001) and fibroblasts (*P* < 0.01). Surprisingly, the TGF-β/SMAD2 inhibitor SB431542 significantly inhibited the effect of HuMSC-EV and reduced the kinetic of wound closure in endothelial cells (*P* < 0.001) but not in fibroblasts (see Supplementary Fig. S2 online).

### HuMSC-EV induce mobilization and recruitment of innate inflammatory cells, and promote neovascularization in irradiated tissue

**Fig. 3:**
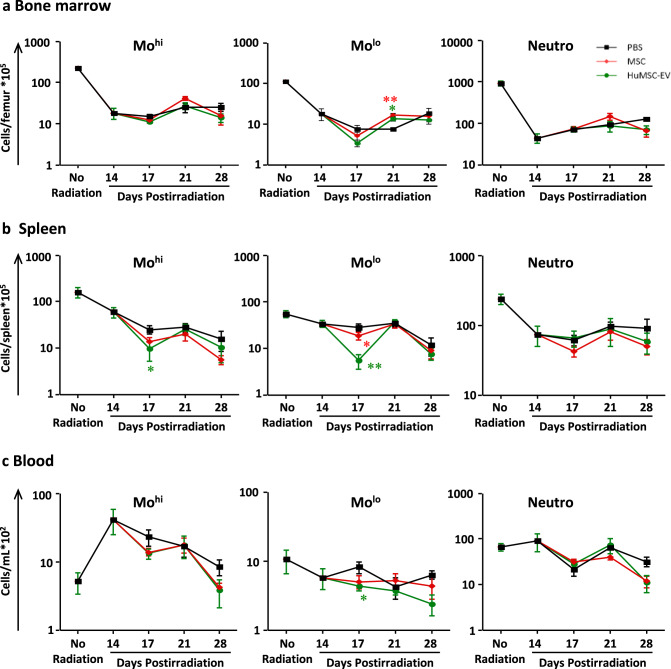
HuMSC-EV induce monocyte mobilization from BM and spleen. Quantitative analysis of cell counts for Mo^hi^ (CD11bLy6G–7/4hi) (left), Mo^lo^ (CD11bLy6G–7/4lo) (middle), gated on CD45+, CD11b+cells, and neutrophils (CD11b+Ly6G+7/4hi) (right) in BM (**a**), spleen (**b**) and blood (**c**) at days 14, 17, 21 and 28 post-irradiation. Data are means ± SEM (*n* = 5–6 animals/group); *P < 0.05, **P < 0.01 between HuMSC-EV and PBS; one-way ANOVA followed by Tukey’s test.

**Fig. 4:**
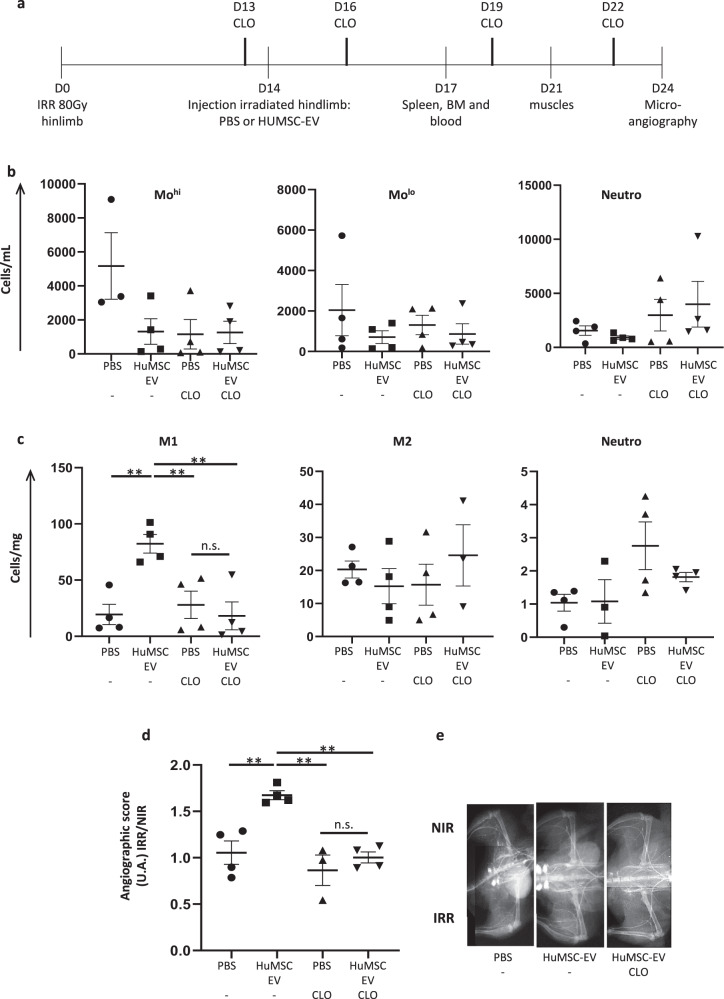
Monocyte recruitment induced by HuMSC-EV participates to the neovascularization process in immunodeficient Nude mice. **a** Experimental design indicating the days of irradiation, HuMSC-EV injection, clodronate treatment, and tissue collection or analysis. **b** Quantitative analysis of cell counts for Mo^hi^ (CD11b+Ly6G–7/4hi) (left), Mo^lo^ (CD11b+Ly6G–7/4lo) (middle), gated on CD11b+ cells, and neutrophils (CD11b+Ly6G+7/4hi) (right) in blood at day 17 post-irradiation. **c** Quantitative analysis of cell counts for M1-like macrophages (CD11b+Ly6G-F4/80+CD64+MHCII+) (left), M2-like macrophages (CD11b+Ly6G-F4/80+CD64+MHCII−) (middle) and neutrophils (right) in irradiated gastrocnemius muscle at day 21 post-irradiation. **d** Quantitative analysis of microangiography at day 24 post-irradiation, represented as the ratio of the angiographic score of the irradiated limb to the angiographic score of non-irradiated limb. **e** Representative pictures of microangiography of the non-irradiated limb (NIR, top) and irradiated limb (IR, bottom), in animals injected with PBS and control liposomes (left), HuMSC-EV and control liposomes (middle) and HuMSC-EV with clodronate containing liposomes (right). Data are means ± SEM (*n* = 4 animals/group); *P < 0.05, **P < 0.01; n.s., not significant; one-way ANOVA followed by Tukey’s test.

BM and spleen are known to release inflammatory cells into the blood after injury. In Nude mice, flow cytometry analysis performed 14 days after irradiation revealed a strong depletion of Mo^hi^ (CD11b^+^Ly6G^-^7/4^hi^), Mo^lo^ (CD11b^+^Ly6G^-^7/4^lo^), and neutrophils in the BM and the spleen, compared to non-irradiated condition (Fig. 3a, b). This effect was maintained until 1 month. Interestingly, HuMSC-EV administration at D14 resulted in a significant 1.8-fold increase of Mo^lo^ in the BM (*P* < 0.05), likely due to cell proliferation, compared to PBS-injected animals at D21 (Fig. 3a). However, no significant variation of Mo^hi^ numbers was observed in the BM after HuMSC-EV injection. More importantly, in the spleen Mo^hi^ and Mo^lo^ cell numbers significantly decreased by 2.6-fold (*P* < 0.05) and 5.2-fold (*P* < 0.01), respectively, at D17 and returned to PBS control levels at later time points (Fig. 3b). In the blood, Mo^hi^ tended to decrease after HuMSC-EV and MSC injection compared to PBS, while Mo^lo^ were significantly decreased by 1.9-fold (*P* < 0.05) at D17 in HuMSC-EV injected animals compared to MSC and PBS group (Figs. 3c, 4a, b). No significant difference was observed between experimental groups for neutrophil numbers in these 3 compartments for all time points.

At D21, HuMSC-EV promoted the recruitment of Mo^lo^ and Mo^hi^ and their differentiation in macrophages in the muscle (*P* < 0.01; Fig. 4c). In our settings, the numbers of type 1-like macrophages (M1) were increased by 4.5-fold in the muscle after HuMSC-EV injection compared to PBS-injected animals (*P* < 0.01; Fig. 4c). These results suggest that administration of HuMSC-EV at the irradiated site induced the recruitment of Mo^hi^ and Mo^lo^ from spleen and BM into the muscle, with a pro-inflammatory potential at early stage of the wound healing process.

Furthermore, we evaluated the arteriogenic process by micro-angiography analysis following HuMSC-EV or PBS administration. We found a significant 1.7-fold increase of the angiographic score at D24 in the irradiated muscle of mice injected with HuMSC-EV compared to PBS-injected animals (*P* < 0.01; Fig. 4d, e). Altogether, the proarteriogenic, proangiogenic and provasculogenic effects described above, suggest a global beneficial effect of HuMSC-EV in the neovascularization process of irradiated tissue.

### Depletion of monocytes/macrophages impairs neovascularization after HuMSC-EV administration

In order to highlight the role of monocytes/macrophages on the neovascularization process, we depleted circulating monocytes by treatment with clodronate-containing liposomes from the day before HuMSC-EV or PBS injection (Fig. 4a). In agreement with previously published data [23], clodronate treatment resulted in a decrease of circulating Mo^hi^ and Mo^lo^ by 70 and 36%, respectively, in PBS-injected control mice (Fig. 4b). In HuMSC-EV-injected animals however, no additional monocyte-depleting effect of clodronate was observed compared to control liposome treatment (Fig. 4b). Nonetheless, administration of clodronate resulted in markedly reduced M1-like differentiation in response to HuMSC-EV injection (*P* < 0.01; Fig. 4c), compared to control liposome treatment, which was associated with a complete abrogation of the beneficial proarteriogenic effect of HuMSC-EV on the neovascularization process (*P* < 0.01; Fig. 4d, e).

### Deficiency in CCR2 compromises HuMSC-EV-induced neovascularization

**Fig. 5:**
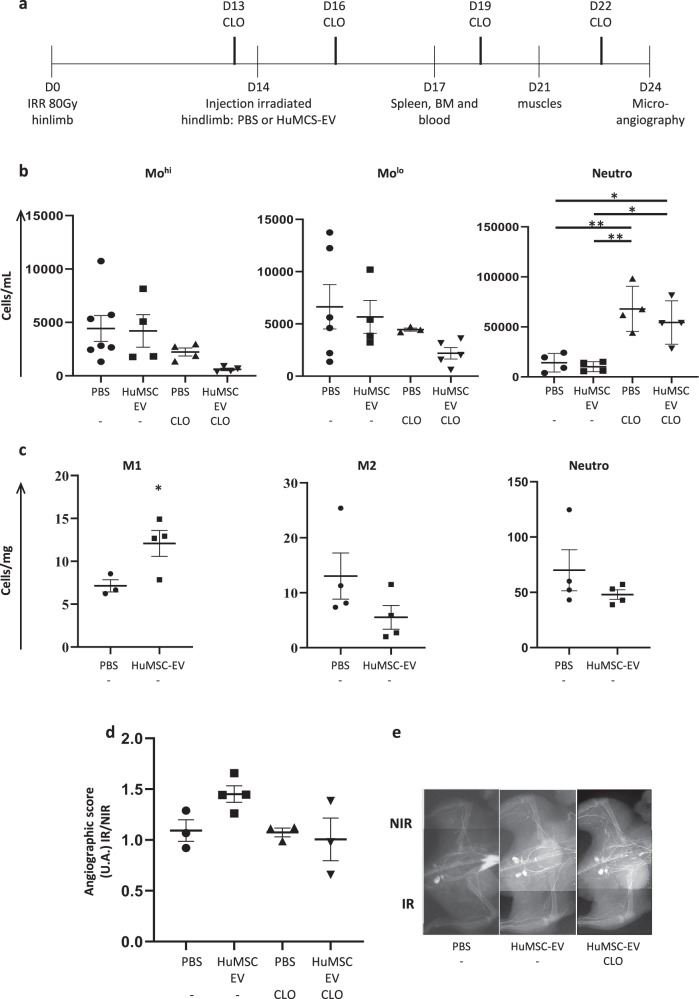
HuMSC-EV-induced monocyte recruitment contributes to the neovascularization process in immunocompetent C57BL/6 mice. **a** Experimental design indicating the days of irradiation, HuMSC-EV injection, clodronate treatment, and tissue collection or analysis. **b** Quantitative analysis of cell counts for Mo^hi^ (CD11b+Ly6G−7/4hi) (left), Mo^lo^ (CD11b+Ly6G−7/4lo) (middle), gated on CD11b+ cells, and neutrophils (CD11b+Ly6G+7/4hi) (right) in blood at day 17 post-irradiation. **c** Quantitative analysis of cell counts for M1-like macrophages (CD11b+Ly6G-F4/80+CD64+MHCII+) (left), M2-like macrophages (CD11b+Ly6G-F4/80+CD64+MHCII−) (middle) and neutrophils (right) in irradiated gastrocnemius at day 21 post-irradiation. **d** Quantitative analysis of microangiography at day 24 post-irradiation, represented as the ratio of the angiographic score of the irradiated limb to the angiographic score of non-irradiated limb. **e** Representative pictures of microangiography of the non-irradiated limb (NIR, top) and irradiated limb (IR, bottom), in animals injected with PBS and control liposomes (left), HuMSC-EV and control liposomes (middle) and HuMSC-EV with clodronate containing liposomes (right). Data are means ± SEM (*n* = 4 animals/group); *P < 0.05, **P < 0.01; Mann−Whitney and one-way ANOVA followed by Tukey’s test.

The effects of HuMSC-EV on the recruitment of innate inflammatory cells and neovascularization were similarly observed in C57BL/6 (immunocompetent) animals (Fig. 5a). Luminex-based measurement of donor-specific type I anti-HLA antibody in different mice sera did not reveal any immune reaction against HuMSC-EV at D7 following their administration (Table 1).

**Table 1:** Anti-HLA analysis in mice sera following injection of HuMSC-EV.

procedure	Sample	Kit neg control	Kit pos control	Lab pos control	#E1	#E2	#E3	#E4	#P1	#P2	#P3	#P4
Standard procedure (10 µL serum vol)	Serum vol	10 µL	10 µL	10 µL	10 µL	10 µL	10 µL	10 µL	10 µL	10 µL	10 µL	10 µL
	Beads vol	40 µL	40 µL	40 µL	40 µL	40 µL	40 µL	40 µL	40 µL	40 µL	40 µL	40 µL
	MFI max	240	20000	7000	138	159	174	148	157	177	166	147
	Test result	NEG	POS	POS	NEG	NEG	NEG	NEG	NEG	NEG	NEG	NEG
Adapted procedure #1 (20 µL serum vol)	Serum vol			20 µL	20 µL	20 µL	20 µL	20 µL				
	Beads vol			40 µL	40 µL	40 µL	40 µL	40 µL				
	MFI max			10000	180	160	152	165				
	Test result			POS	NEG	NEG	NEG	NEG				
Adapted procedure #2 (40 µL serum vol)	Serum vol				40 µL	40 µL	40 µL	40 µL				
	Beads vol				40 µL	40 µL	40 µL	40 µL				
	MFI max				175	170	160	160				
	Test result				NEG	NEG	NEG	NEG				

The Luminex-based LSA assay resulted in negative response in all animals tested using the standard procedure with 10 µL serum volume per reaction. The procedure was repeated using increasing amounts of starting serum volume up to 40 µL to ascertain the negativity of the tests. An MFI (mean fluorescence intensity) value of >1500 was considered positive. #EV1, HuMSC-EV-injected animal ID; #P1, PBS-injected animal ID; neg, negative; pos, positive.

While circulating Mo^hi^ and Mo^lo^ numbers were not changed upon HuMSC-EV injection (Fig. 5b), M1-like cell numbers were significantly increased by 2.1-fold in the muscle following HuMSC-EV injection compared to PBS injection at D21 (*P* < 0.05; Fig. 5c). In addition, a trend toward an increase of the angiographic score in the irradiated muscle after HuMSC-EV administration was observed, compared to PBS-injected animals (Fig. 5d, e).

Clodronate treatment resulted in a decrease of circulating Mo^hi^ and Mo^lo^ by 50 and 33%, respectively, compared to control liposome-injected C57BL/6 mice (Fig. 5b). Similar to Nude mice, the beneficial proarteriogenic effect of HuMSC-EV on the neovascularization process was completely abrogated by administration of clodronate in C57BL/6 mice (Fig. 5d, e).

**Fig. 6:**
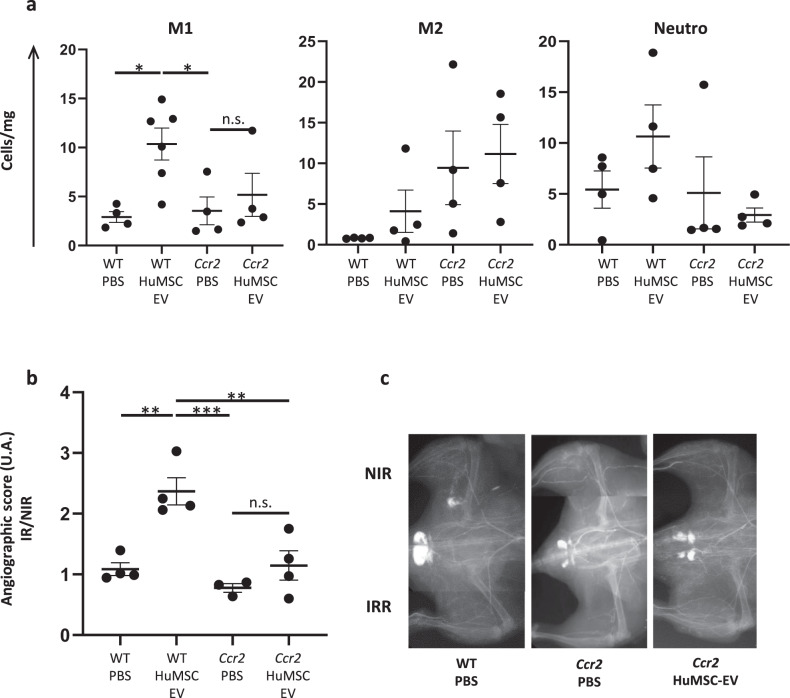
CCR2 deficiency abrogates the effect of HuMSC-EV on monocyte recruitment and neovascularization process. **a** Quantitative analysis of cell counts for M1-like macrophages (CD11b+Ly6G-F4/80+CD64+MHCII+) (left), M2-like macrophages (CD11b+Ly6G−F4/80+CD64+MHCII−) (middle) and neutrophils (right) in irradiated gastrocnemius, at day 21 post-irradiation. **b** Quantitative analysis of microangiography at day 28 post-irradiation, represented as the ratio of the angiographic score of the irradiated limb to the angiographic score of non-irradiated limb. **c** Representative pictures of microangiography of the non-irradiated limb (NIR, top) and irradiated limb (IR, bottom), in WT-PBS (left) and Ccr2-PBS (middle), and Ccr2-HuMSC-EV (right). Data are means ± SEM (*n* = 4 animals/group); *P < 0.05, **P < 0.01, ***P < 0.001; n.s., not significant; one-way ANOVA followed by Tukey’s test.

Given that clodronate treatment non-specifically depletes all circulating monocyte subtypes, we investigated more precisely the role of M1-like cells in HuMSC-EV-mediated effect on neovascularization by using CCR2-deficient mice (Fig. 6). Strikingly, the HuMSC-EV-mediated increase in M1-like cell numbers observed in the muscle of WT animals (*P* < 0.05 compared to PBS; Fig. 6a) was completely abrogated in *Ccr2*^*−/−*^ mice. Accordingly, a 2-fold reduction of the angiographic score was found in *Ccr2*^*−/−*^ mice injected with HuMSC-EV, compared to WT mice (*P* < 0.01; Fig. 6b, c).

Our findings clearly identify an early key role for monocyte/macrophage activation via CCL2/CCR2 signaling, in the organization of vascular inflammation and the stimulation of neovascularization after HuMSC-EV administration at the radiation injury site.

## Discussion

MSC have been shown to secrete paracrine factors that stimulate neovascularization [6, 24, 25]. In this study, we demonstrated that the proangiogenic paracrine activity associated with MSC is mediated by EV. We showed, for the first time, that monocytes/macrophages contributed to the neovascularization process induced after injection of HuMSC-derived EV in radiation-injured tissue.

We have demonstrated that ionizing radiation induces acute changes in basement membranes, increases vascular permeability, and reduces neovascularization, thereby affecting the wound healing process [4–6]. We evidenced that irradiation hampered skin perfusion, vascular density, mobilization of endothelial progenitors and immune cells, and production of proangiogenic factors.

We, therefore, assessed the effect of HuMSC-EV on new blood vessel formation, as activation of angiogenesis, arteriogenesis, and vasculogenesis is necessary for tissue regeneration. In our model, we demonstrated that HuMSC-EV increased blood perfusion, angiographic score and capillary density compared to PBS-injected animals. We have also demonstrated an increase of HIF-1α, VEGF, eNOS and MCP-1/CCL2 expression in the injured tissue following HuMSC-EV administration. These results suggest that HuMSC-EV induce local overexpression of proangiogenic factors, although we cannot exclude a direct contribution of intravesicular material, since it was shown that HuMSC-EV contains growth factors, cytokines, signaling molecules, including the proangiogenic factors VEGF, TGF-β1, and IL-8 [26]. Many studies have shown that these proangiogenic factors are critical regulators of angiogenesis and arteriogenesis in wound healing [27–29]. We demonstrated that, during the regenerative phase, HuMSC-EV injection led to increased expression levels of the proangiogenic factors HIF-1α, VEGF, and eNOS, likely in order to contribute to the recruitment, mobilization, adhesion and migration of BM progenitor cells to the injured tissue.

The activation of the HIF system must be very finely regulated in order to avoid any harmful effects. HIF itself activates its own degradation system, by increasing the expression of PHD2 through an hypoxia-response element located in its promoter [30]. PHD2 is induced gradually during hypoxia [31], and PHD2 protein levels were increased in tissues from HuMSC-EV-injected animals, where HIF-1α was increased.

Similarly, HuMSC-EV increased the level of Peroxiredoxin1 and SOD1 expression in the muscle, which are known to decrease ROS production, dramatically enhanced after ionizing radiation exposure. Overproduction of ROS contributes to the impairment of post-ischemic neovascularization [32]. Using in vitro model, we investigated the molecular mechanisms involved in the beneficial effect of HuMSC-EV in both endothelial cells and fibroblasts cell types. Furthermore, we observed that this beneficial effect on wound closure depends on both PI3K/AKT and TGF-β/SMAD2 pathways, for endothelial cells and specifically on the PI3K/AKT pathway on fibroblasts, as previously described in other settings [33].

In addition, HuMSC-EV contributes to the regeneration of skeletal muscle. We found that miR-133 and miR-206 were increased after HuMSC-EV injection, and the local injection of these miRNAs has been shown to stimulate muscle regeneration in a rat skeletal muscle injury model [34]. Recently, a study has demonstrated the effects of EV secreted by cardiac mesenchymal stem cells (C-MSC-EV) in protecting the myocardium from reperfusion injury and identified mmu-miR-7116-5p as the most abundant miRNA in C-MSC-EV [35]. These findings may open new avenues of research for EV in optimizing future stem cell-based therapy in regenerative medicine [36].

As previously described, the wound healing process involves two distinct phases where inflammation plays a key role. The regenerative phase is characterized by Mo^hi^ and Th1 phenotype, associated with the substitution of injured cells by cells of the same type and neovascularization process [15, 37]. The second stage is associated with Mo^lo^ and Th2 activation and the formation of permanent scar. We observed, in the regenerative phase, that the neovascularization process was associated with an increase of pro-inflammatory Mo^hi^ and M1-like cells. MSC-derived EV have been shown to promote myeloid-biased multipotent hematopoietic progenitor expansion via TLR4 activation-canonical NF-κB-HIF-1α-CCL2 signaling [38]. Thus, we explored the immune modulation after HuMSC-EV administration. In fact, the depletion of circulating monocytes following clodronate treatment, and more specifically the strong reduction of circulating Mo^hi^/M1-like cells in *Ccr2*^*-/-*^ mice, abrogated the neovascularization process induced by HuMSC-EV injection. We can conclude that, in our model, Mo^hi^/M1-like cells participate to the innate system to improve the neovascularization process. These results are in accordance with previously published data showing that CCL2/CCR2-mediated inflammatory response is essential to repair acute skeletal muscle injury. Indeed, *Ccr2*^-*/*-^ or *Ccl2*^-*/*-^ mice displayed reduced macrophage infiltration in response to acute muscle injury [39, 40], and this effect was associated with a decrease in muscle regeneration [41]. However, other chemoattractant pathways might be involved, such as CX3CL1/CX3CR1, which promotes Mo^hi^ and Mo^lo^ infiltration into atherosclerotic plaques [42], and CX3CR1 controls Mo^lo^ infiltration into the ischemic myocardium [15]. In addition, a previous study highlighted the involvement of CCL2/CCR2 signaling in monocyte mobilization from BM [43].

In conclusion, our study demonstrates, for the first time, that HuMSC-EV administration improved the neovascularization process of irradiated tissues. HuMSC-EV administration resulted in increased production of proangiogenic factors, and enhanced vasculogenesis and arteriogenesis. Moreover, we showed that monocytes/M1-like cell mobilization and recruitment participate to the early phase of vessel growth after hindlimb irradiation.

## Material and methods

### Study design

This study was designed to investigate the therapeutic efficiency of HuMSC-EV in a murine model of radiation injury, and to decipher the mechanisms involved in EV-induced neovascularization. HuMSC-EV beneficial effect was investigated in immunocompromised, immunocompetent, and Ccr2 knockout mouse model systems, strongly suggesting HuMSC-EV provascular effect in the irradiated tissue is mediated by monocytes/macrophages. All animal procedures were approved by the institutional animal experimentation and ethics committee (C2EA) at the Institute of Radiation protection and Nuclear Safety (IRSN). Experiments were conducted according to the French veterinary guidelines and those formulated by the European Community for experimental animal use (approval APAFIS#9773-201704271703979 vI) and were carried out in accordance with the ARRIVE guidelines. All mice were included in the study, were kept under stable environment conditions (22 ± 1 °C), with alternating 12 h light and dark cycles and received standard laboratory food and water. Animals were monitored to establish a semi quantitative injury score, based on the assessment of wound extent, ulceration, moist desquamation and limb retraction during the wound healing process from D14 to D28 after irradiation. Treatment groups were constituted based on similar injury scores. Blinding was not possible in most animal experiments.

Power analysis was computed using InVivoStat algorithm [44]. The number of samples was estimated to be sufficient to provide statistical power of at least 80% needed to obtain a *P* value of less than 0.05 for injury score and neovascularization assessment in between group comparisons, and to account for multigroup post hoc testing. A sample size of *n* ≥ 10 per group was thus calculated to allow us to estimate HuMSC-EV beneficial effect for the treatment of radiation injury in Nude mice. Mechanistic experiments were conducted on *n* = 6 animals per group in order to obtain statistically significant results regarding the vascular parameters under study. Experiments with clodronate treatment and *Ccr2*^*−/−*^ mice were performed on *n* = 4 animals per group.

### Mice and irradiation

Male Nude and C57BL/6 mice (8 weeks old) were used as experimental animals (Janvier, France). Eight-week-old male *Ccr2*^*−/−*^ mice and their wild-type (WT) C57BL/6 littermates were purchased from the Jackson Laboratories. All animals were acclimated in the animal research facility at IRSN before irradiation at the age of 10 weeks. Mice were anesthetized by isoflurane inhalation and their right hindlimb was exposed to a single dose of 80 Gy irradiation using an Elekta Synergy Platform delivering 10-MV X-rays at 2.6 Gy/min (Elekta SAS, Boulogne-Billancourt, France). For chimerism assay, 10–12 weeks old GFP+ C57BL/6 mice were used as bone marrow donors (Jackson laboratories, Bar Harbor, ME). Recipient C57BL/6 mice (10 weeks old, Janvier) were exposed to 10 Gy whole body irradiation the day prior to GFP+ bone marrow transplant.

### HuMSC-EV therapeutic effect

At D14 post-irradiation, three groups of Nude mice (*n* = 36 animals/group, 3 independent experiments) were constituted with equivalent scoring for intramuscular injection of either 10^6^ human adipose tissue-derived MSC (collaboration JJ Lataillade, CTSA, Clamart, France), 400 μg of EV isolated from human ES-derived MSC [45, 46], or PBS at the radiation injured site. Animals were euthanized by cardiac puncture followed by cervical dislocation at different time points, i.e., D17 (*n* = 36/group), D21 (*n* = 27/group), D24 (*n* = 27/group), and D28 (*n* = 18/group). Blood, spleen, bone marrow, tibialis and gastrocnemius muscles were harvested for analysis.

### HuMSC-EV characterization

The isolation and characterization of EV from human ES-derived MSC was reported in detail in previous publications [19, 26, 45–48].

### Experimental approach to investigate the effect of HuMSC-EV on the neovascularization process

Neovascularisation was evaluated by three different methods as previously described [49, 50].

#### Laser Doppler perfusion imaging

To provide functional evidence for changes in neovascularization after injections, laser Doppler (Moor instruments, Devon, UK) perfusion imaging analyses were performed as previously described on both irradiated and non-irradiated limbs at D17, D21 and D28 [51]. Blood perfusion is expressed as the irradiated/non-irradiated limbs ratio.

#### Capillary density measurement

Mice were anesthetized by isoflurane and euthanized. The gastrocnemius tissue was excised from both irradiated and non-irradiated limbs, and then snap frozen in O.C.T. compound (Sakura Finetek France, Villeneuve d’ASCQ, France) for cryosectioning. Frozen muscle sections (7 µm) were stained using a mouse-specific CD31 antibody (cat# 550274, BD Biosciences, Le Pont de Claix, France), followed by Alexafluor-594 secondary antibody (cat# A-21209, Thermo Fisher Scientific, Illkirch, France). For quantification, the numbers of CD31-positive cells were counted in 10 randomly selected transverse sections in each animal. The results were averaged, and capillary density was expressed as a ratio of the number of CD31-positive cells per mm^2^ field in irradiated muscle to non-irradiated muscle.

#### Microangiography

Mice were anesthetized by pentobarbital injection and a longitudinal laparotomy was performed to introduce a polyethylene catheter into the abdominal aorta and inject barium sulfate contrast medium (1 g/mL, cat# B8675, Sigma-Aldrich, St Quentin Fallavier, France). Images were acquired with the use of a high-definition digital X-ray transducer (Kodak, RVG 5100). Vessel density was expressed as a percentage of pixels per image occupied by vessels in the quantification area.

### Bone marrow transplantation and mobilization potential

WT recipient C57BL/6 mice were anesthetized with isoflurane inhalation and a single dose of 10 Gy was delivered in a whole-body configuration. On the next day, BM cells were harvested from 10 to 12 weeks old non-irradiated GFP‐C57BL/6 mice, and mononuclear cells were purified by density centrifugation using Ficoll gradient (Histopaque-1083, cat# 10831, Sigma-Aldrich) (25 min, 400 × *g*, room temperature). Isolated BM cells were administered by retro-orbital injection into lethally irradiated C57BL/6 (C57BL/6-GFP→C57BL/6) animals (5 × 10^6^ cells per mouse). Eight weeks later, blood cell chimerism was confirmed by flow cytometry, and chimeric mice underwent hindlimb irradiation followed by HuMSC-EV or PBS injection, as described above. The protein expression of green fluorescent protein (GFP) in irradiated tissue of chimeric mice was analyzed by Western blot and immunostaining 3 and 5 days after injection. Additionally, frozen muscle sections (7 µm) were immunostained using a mouse-specific CD31 (BD Biosciences) followed by Alexafluor-594 secondary antibody (Thermo Fisher Scientific).

### Experimental approach to investigate the effect of HuMSC-EV and the role of monocytes/macrophages in neovascularization

A series of experiments with similar analytical endpoints was performed on independent groups of Nude, WT C57BL/6, and *Ccr2*^*−/−*^ mice (Figs. 4, 5, and 6). Animals were locally injected with HuMSC-EV or PBS 14 days after hindlimb irradiation, as described above. In order to explore the proangiogenic and proarteriogenic potential of monocytes/macrophages, circulating monocytes were depleted in Nude or WT C57BL/6 mice by treatment with liposomes containing clodronate (Cat# CP-025, dichloromethylene diphosphonate; ClodronateLiposomes.org, Amsterdam, Netherlands), starting from the day before HuMSC-EV injection. Animals received a retro-orbital injection of 150 μl of PBS-control liposomes or clodronate-loaded liposomes every 3 days for 10 days.

Innate immune cell mobilization was monitored at D17 (3 days after HuMSC-EV or PBS injection) by flow cytometry, macrophage recruitment in irradiated tissues was analyzed at D21, and neovascularization was investigated by micro-angiography at D24.

### Immunological analyses

Detection of donor-specific anti-human leukocyte antigen (HLA) antibodies was performed using the Luminex single antigen (LSA) technology. The Lifecodes LSA Class I (Cat# 265 100, Immucor, Norcross, GA) determined the specificity of class I HLAs in A/B IgG antibodies in 10 µL mouse serum, according to the manufacturer’s instructions. In order to confirm the absence of rejection, the test was repeated using increasing volumes up to 40 µL of serum. The presence and specificity of antibodies were then detected on a Luminex 100 IS flow analyzer (Luminex, Canoga Park, CA), and the mean fluorescence intensity (MFI) for each sample in each bead was evaluated. An MFI value of >1500 was considered positive.

### Statistical analysis

All data are expressed as median and range (25th percentile and 75th percentile). Graphs represent the distribution of the populations with median (horizontal bar), 25th and 75th percentile (boxes), and 10th and 90th percentile (error bar).

Analyses were performed using two-tailed Mann–Whitney or one-way analysis of variance (ANOVA) followed by Tukey’s multiple comparison test using GraphPad Prism software v.5.03 (GraphPad Software, San Diego, CA). Results with a *P*-value <0.05 were considered significant. Estimate of variant was not performed prior to any statistical analyses. The variance was similar in all comparison groups.

## Supplementary information

Loinard_suppl data

Original Data File

Figure S1

Figure S2

## Data Availability

All data generated or analyzed during this study are included in this published article (and its Supplementary Information files).
