# Supplementary material for: HuMSC-EV induce monocyte/macrophage mobilization to orchestrate neovascularization in wound healing process following radiation injury
Source: Cell Death Discov. 2023 Feb 1;9:38. doi: 10.1038/s41420-023-01335-y (PMC9892506; doi:10.1038/s41420-023-01335-y)
Supplement: Supplementary file 1 — Loinard_suppl data [file 41420_2023_1335_MOESM1_ESM.pdf]

HuMSC-EV induce monocyte/macrophage mobilization to orchestrate neovascularization in wound healing process following radiation injury

Céline Loinard, PhD, Alexandre Ribault, PhD, Bruno Lhomme, Marc Benderitter, PhD, Stéphane Flamant, PhD, Sandrine Paul, Valérie Dubois, Ruenn Chai Lai, PhD, Sai Kiang Lim, PhD, and Radia Tamarat, PhD*.

Supplementary data

Supplementary Materials and Methods

Scratch wound assay

Human Dermal Microvascular Endothelial Cells (HMVEC-d, cat# CC-2543, Lonza, Verviers, Belgium) were cultured in EBM-2 medium supplemented with 0.1% hEGF, 0.04% hydrocortisone; 0.1% GA-1000, 20% FBS, 0.1% VEGF, 0.4% hFGF-B, 0.1% R3-IGF-1 and 0.1% ascorbic acid (cat# CC-4176, Lonza). Normal Human Dermal Fibroblasts (NHDF-Ad, CC-2511, Lonza) were cultured with FBM-2 medium supplemented in 2% FBS, 0.1% insulin, 0.1% hFGF-B and 0.1% GA-1000 (CC-3132, Lonza). After thawing, all cells were cultured in 75cm² flasks for amplification, then subcultured in 12-well plates by using Trypsin-EDTA (1X) 0.05% (cat# R001100, Thermo Fisher Scientific, Illkirch, France). Experiments were performed on cultures at passage 5.

A scratch injury model was used to investigate the mechanisms of action of EV on the wound healing process. After reaching confluence state, cells were exposed to 20-Gy irradiation and wounds were mechanically made using pipette tip. Cells were washed twice with PBS, and fresh medium supplemented with 50µg of EV, alone or in combination with 10µM of PI3K/AKT inhibitor (LY294002, cat# L9908, Sigma-Aldrich) or TGF-β/SMAD2 inhibitor (SB431542, cat# S4317, Sigma-Aldrich). Cells were incubated and monitored for 48h using a live-cell analysis

system (Incucyte® S3, Essen BioScience, Ltd, UK). Pictures of cell cultures were taken every 4h and the area of the wound was analysed using Fiji Software [1].

Western blot

Preparation of protein extracts was performed as previously described [2]. Briefly, to prepare total protein, *tibialis anterior* muscles from irradiated and non-irradiated hindlimbs were homogenized in RIPA buffer (50mM Tris HCl pH7.4, cat# T8524, 150mM NaCl, cat# S9888, 1mM EDTA, cat# ED4SS, 1% Triton X-100, cat# T8787, 1% Deoxycholate cat# D6750, 0.1% SDS, cat# 11667289001, all from Sigma-Aldrich, with protease and phosphatase inhibitors, cat# A32965, Thermo Fisher Scientific). To prepare nuclear protein extracts, gastrocnemius muscles from irradiated hindlimb was homogenized in 10mM Tris-HCl pH7.8, 1.5mM MgCl2 (cat# M8266), 10mM KCl (cat# P3911), 0.5mM DTT (cat# 10708984001), 1mM sodium orthovanadate (cat# S6508), all from Sigma-Aldrich, supplemented with 1x protease inhibitor cocktail. After centrifugation at 4500 x *g* for 5 min at 4°C, pellets were resuspended at 4°C for 30 min in 20mM Tris-HCl pH7.8, 1.5mM MgCl2, 420mM KCl, 20% glycerol, supplemented with 1x protease inhibitor cocktail, and then centrifuged at 10000 x *g* for 30 min, 4°C. Supernatants were dialyzed with Slide-a-Lyzer MINI Dialysis Units (Thermo Fisher Scientific) for 2 hours, twice, at 4°C, in 20mM Tris-HCl pH7.8, 1.5mM MgCl2, 100mM KCl, 0.2mM EDTA and 20% glycerol. Proteins were resolved in gradient denaturing gel electrophoresis and blotted onto 0.2µm nitrocellulose sheets (Biorad, Marnes la Coquette, France). Antibodies against gp91 (cat# ab80508), superoxide dismutase (SOD)1 (cat# ab13498), SOD2 (cat# ab13533), Peroxiredoxin (Perox)1 (cat# ab41906), Perox3 (cat# ab73349), (all at 1:1,000; Abcam, Cambridge, UK), VEGF (cat# A3113, 1:1,000; Santa Cruz Biotechnology), eNOS (cat# 610297, 1:1000; BD Biosciences), PHD2 (cat# MAB76801), PHD3 (cat# MAB6954), HIF-1α (cat# MAB1536) (all at 1:1,000 Bio-Techne, Rennes, France) and PHD1 (cat# A32338, 1:1,000 antibodies.com, Cambridge, UK) were used for immunoblotting. As a protein loading control, membranes were stripped, stained with ponceau red or incubated with a monoclonal antibody

directed against GAPDH (cat# ab8245, 1:10,000, Abcam) and specific chemiluminescent signal was detected as previously described [2].

Real-Time Quantitative Polymerase Chain Reaction

Total RNA and miRNA were extracted from frozen gastrocnemius with mirVana™ miRNA isolation kit (cat# AM1560, Thermo Fisher Scientific) according to the manufacturer's instructions and quantified on a NanoDrop ND-1000 apparatus (NanoDrop Technologies Inc., Rockland, DE). Reverse transcription of miRNA and total RNA was performed using the TaqMan® MicroRNA reverse Transcription kit (cat# 4366596) and the High Capacity Reverse Transcription Kit (cat# 4368814), respectively, according to the manufacturer's instructions (Thermo Fisher Scientific). Quantitative (q)PCR was performed using predeveloped TaqMan® Gene Expression Assays (cat# 4440886, Thermo Fisher Scientific) on an ABI Prism 7900 sequence detection system (Thermo Fisher Scientific). Mouse U6 and GAPDH were used to normalize sample amplification.

Flow cytometry

Mice were euthanized at different times (0, 3, 7, 10, or 14 days) after HuMSC-EV or PBS injection. Peripheral blood was drawn via retro-orbital puncture with heparin solution. Peripheral blood was lysed after immunofluorescence staining using the FACS lysing solution (cat# 349202, BD Biosciences). BM cells were drawn from femur and filtered through a 40-μm nylon mesh (VWR, Fontenay sous Bois, France). Spleens were collected, gently passed through a 40-μm nylon mesh (VWR). For both splenocytes and BM-derived cells, the cell suspension was centrifuged at 400 x *g* for 10 min at 4°C. Red blood cells were lysed using red blood cell lysing buffer (cat# R7757, Sigma-Aldrich) and splenocytes and BM cells were washed with PBS.

Muscles were minced with fine scissors, and gently passed through the Bel-Art Scienceware 12-well tissue disaggregator (Thermo Fisher Scientific). Cells were smashed through a 40μm cell strainer (VWR) and harvested in a 50 mL Falcon conical tube. After centrifugation at 400

x *g* for 15 min at 4°C, cells were resuspended in 100µL of PBS. The total number of cells was then normalized to muscle weight.

Cells were labeled with AF700-conjugated anti-CD45 (30-F11, cat# 1933401, Thermo Fisher Scientific), FITC-conjugated anti-CD11b (M1/70, cat# 1989138, Thermo Fisher Scientific), APC-conjugated anti-Ly-6B.2 (clone 7/4, cat# 1804, Biorad), PE-conjugated anti-Ly6G (1A8, cat# 127612, Biolegend), PB-conjugated anti-CD64 (X54-5/7.1, cat# 139309, Biolegend), APC-conjugated anti-F4/80 (MCA497, cat# 1608, Biorad), PerCP5.5-conjugated anti-MHCII (M5/114.15.2, cat# 107626BD, Biosciences), or isotype controls 488-AF–anti-rat, (cat# 712-545-153) and anti-rabbit IgG (cat# 712-545-152) (Jackson ImmunoResearch, Ely, Cambridgeshire, UK) during 30 min at 4°C. Mo^{hi} ($CD11b^{+}Ly6G^{-}7/4^{hi}$), Mo^{lo} ($CD11b^{+}Ly6G^{-}7/4^{lo}$), M1-like cells ($CD11b^{+}Ly6G^{-}F4/80^{+}CD64^{+}MHCII^{+}$) and M2-like cells ($CD11b^{+}Ly6G^{-}F4/80^{+}CD64^{+}MHCII^{-}$) were gated on $CD45^{+}$ and $CD11b^{+}$ cells. Cells were washed, resuspended in 100µL of PBS and analyzed using a Canto II flow cytometer (BD Biosciences).

Supplementary Figure legends

Supplementary Figure S1 - Analysis of PHD1 and PHD3 protein levels in irradiated gastrocnemius. Representative Western blot and quantification of (a) PHD1 and (b) PHD3 protein levels in animal groups injected with PBS or EV, 3 days after injection.

Supplementary Figure S2 - HuMSC-EV stimulate wound healing process in vitro. Scratch wound assays were performed and monitored for 48h on irradiated dermal endothelial cells and fibroblasts.

(a-b) Representative images of wounds at T0, T0+12h and T0+24h after scratch on dermal endothelial cells (a) and fibroblasts (b).

(c-d) Effect of HuMSC-EV on wound closure by endothelial cells (c) and fibroblasts (d) compared to PBS.

(e-f) Effect of PI3K/AKT inhibitor LY294002 (e) and TGF-β/SMAD2 inhibitor SB431542 (f) on HuMSC-EV-induced wound healing. Data are means ± SEM (2 independent experiments with triplicate data points, *P<0.05, **P<0.01, ***P<0.001).

Supplementary data – full-length Western blots. Full-length Western blots and annotations are presented as supplementary data at the end of this document. Red arrows indicate the wells and bands that were selected to generate the representative Western blot panels in main figures 1 & 2.

Supplementary References

1. Schindelin J, Arganda-Carreras I, Frise E, Kaynig V, Longair M, Pietzsch T, et al. Fiji: an open-source platform for biological-image analysis. Nature methods. 2012;9(7):676-82. Epub 2012/06/30.

2. Loinard C, Ginouves A, Vilar J, Cochain C, Zouggari Y, Recalde A, et al. Inhibition of prolyl hydroxylase domain proteins promotes therapeutic revascularization. Circulation. 2009;120(1):50-9. Epub 2009/06/24.
