# Supplementary figures and images for: HuMSC-EV induce monocyte/macrophage mobilization to orchestrate neovascularization in wound healing process following radiation injury
Source: Cell Death Discov. 2023 Feb 1;9:38. doi: 10.1038/s41420-023-01335-y (PMC9892506; doi:10.1038/s41420-023-01335-y)

Fig 1h

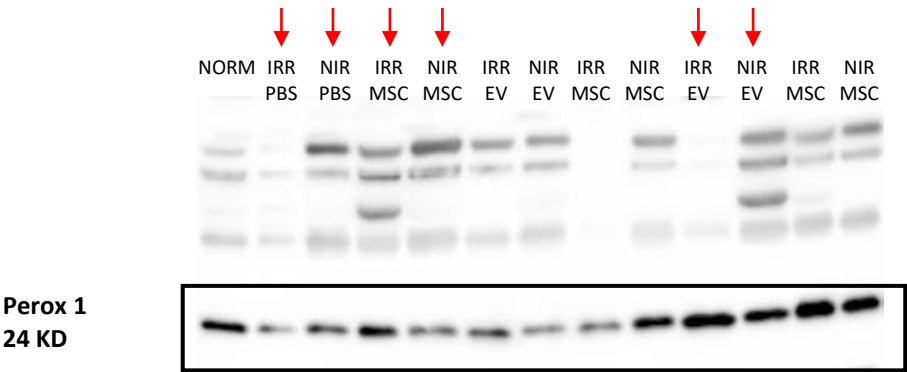

Fig 1j

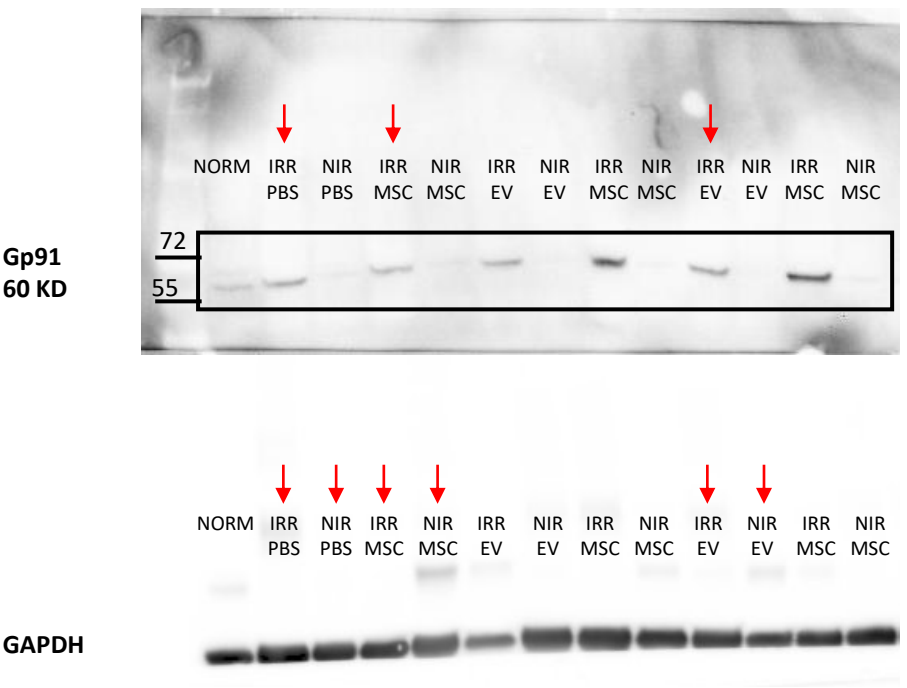

Fig 1i

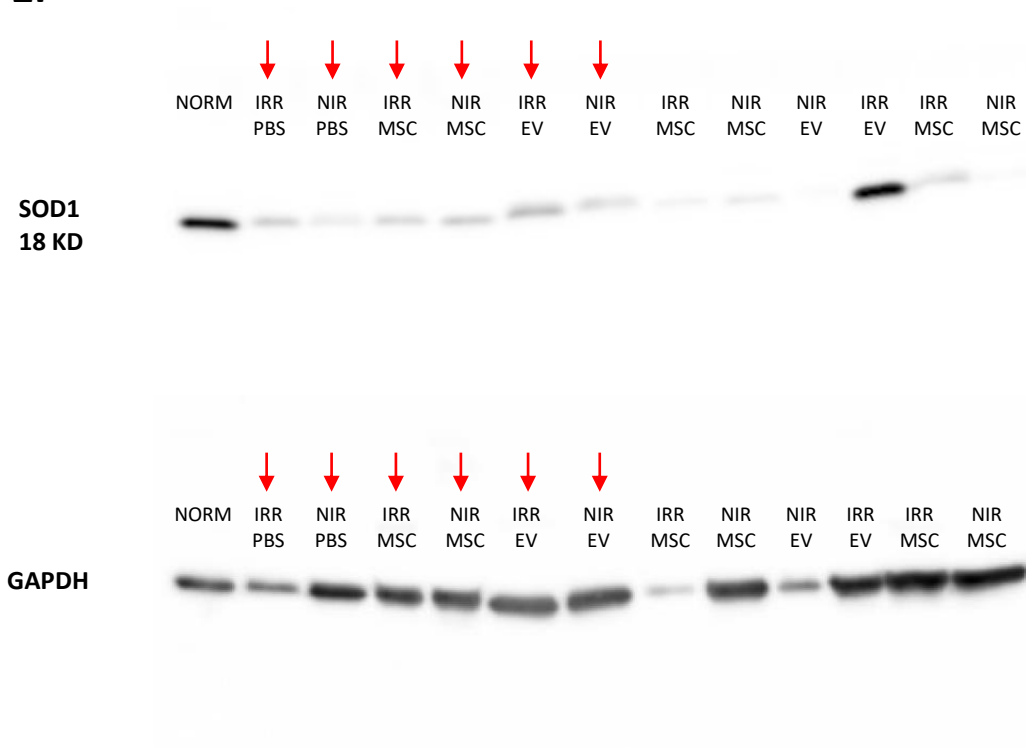

Fig 2c

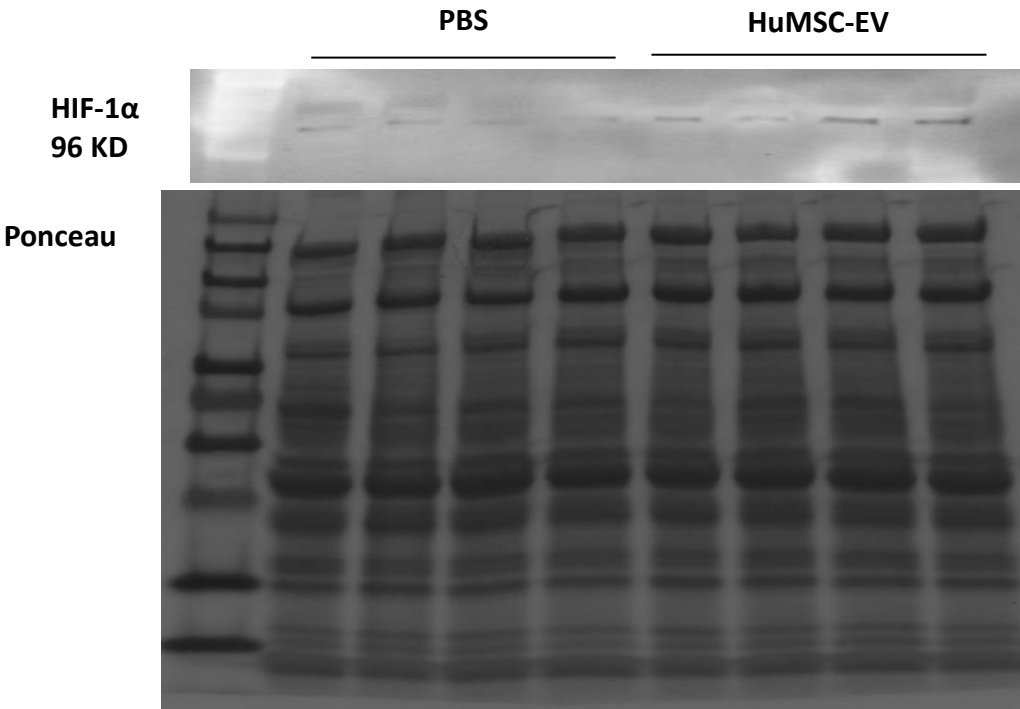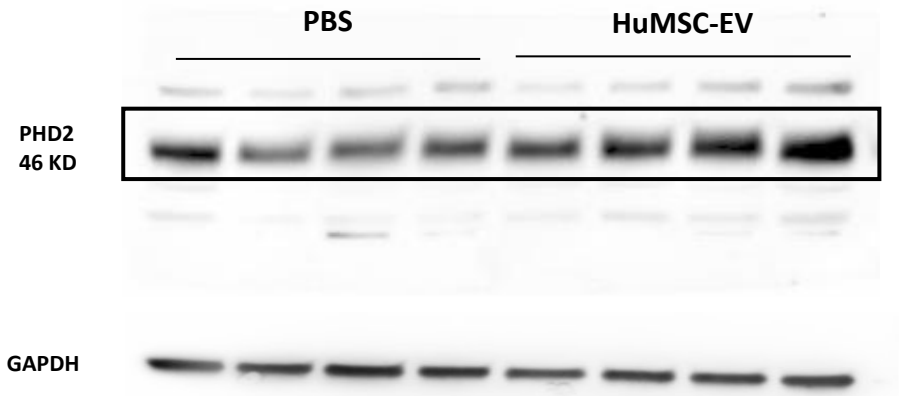

Fig 2d

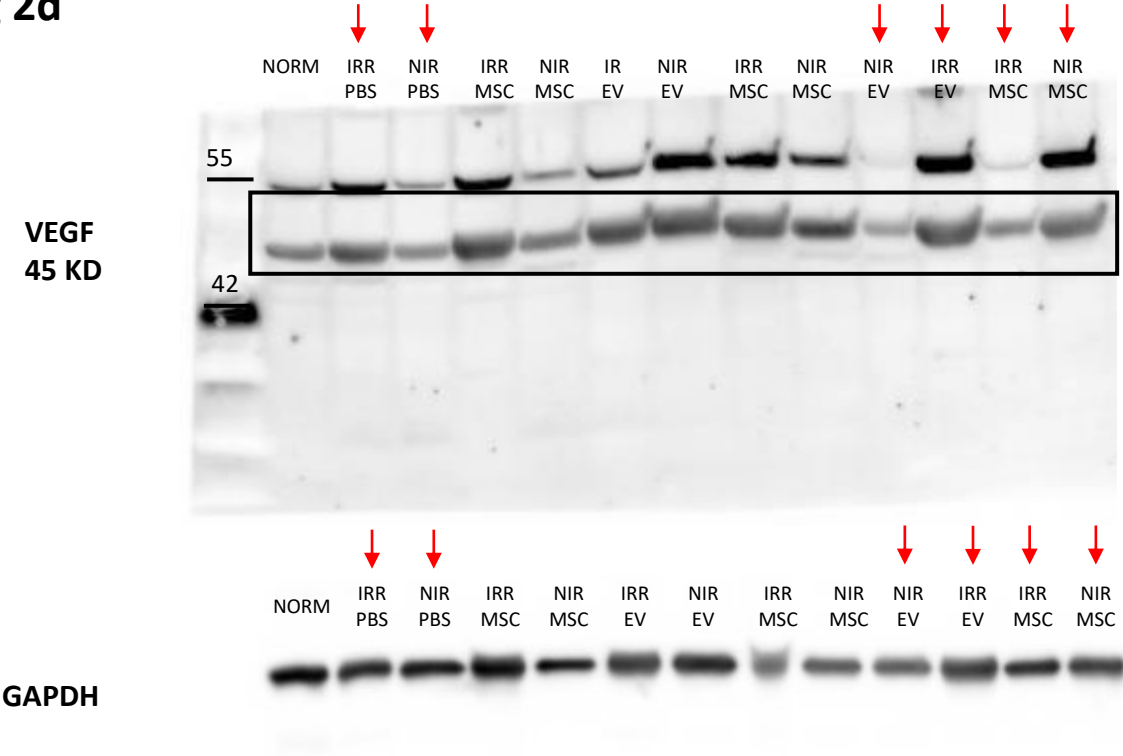

Fig 2e

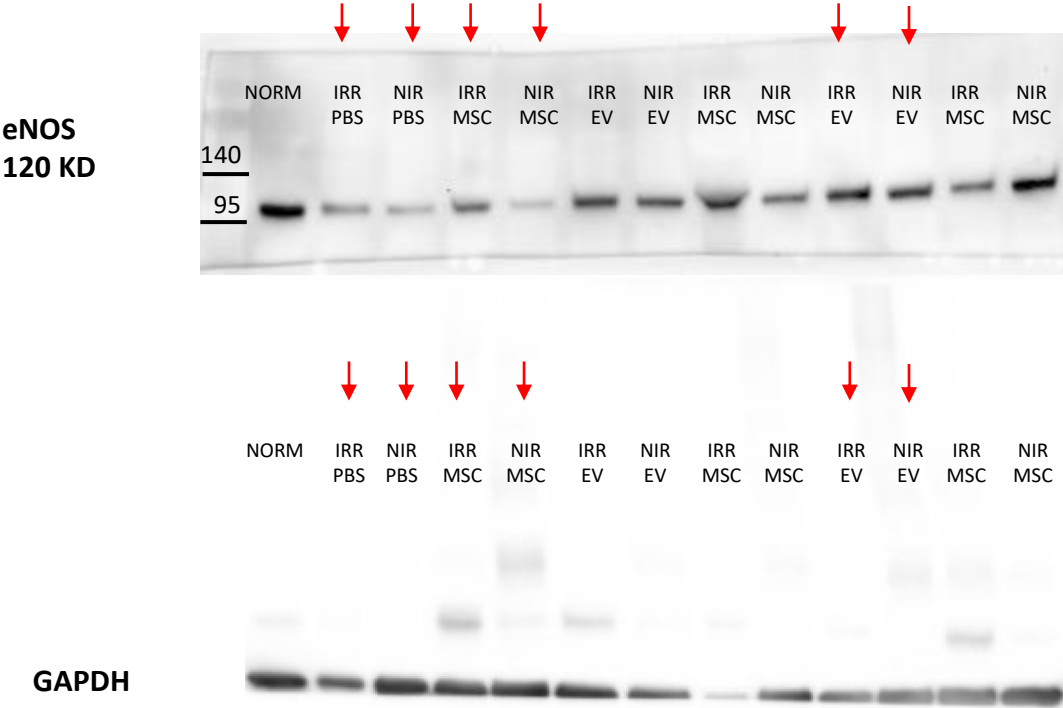

Fig S1

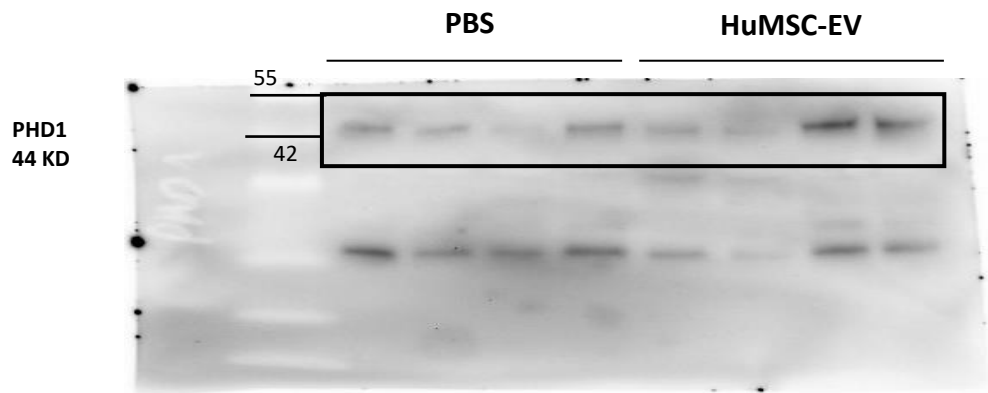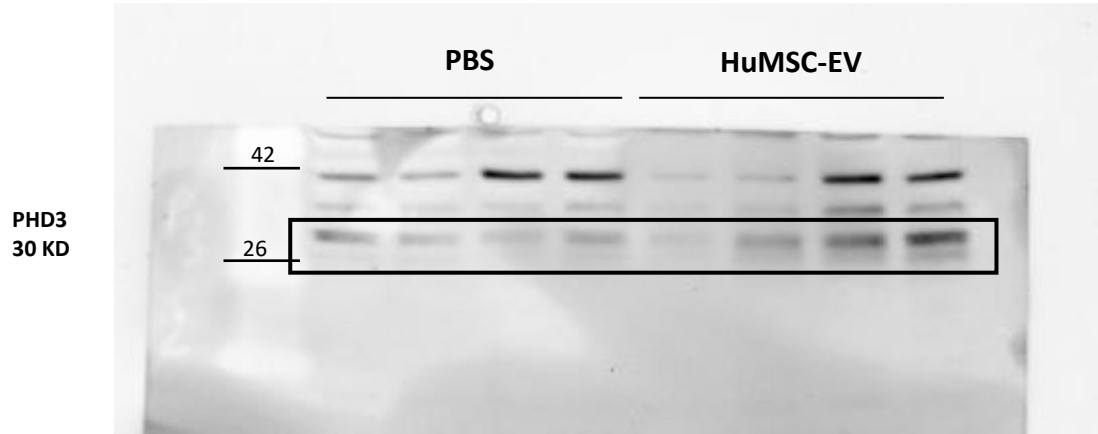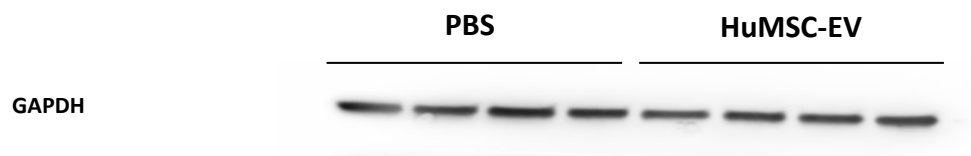

Supplement: Supplementary file 2 — Original Data File [file 41420_2023_1335_MOESM2_ESM.pdf]

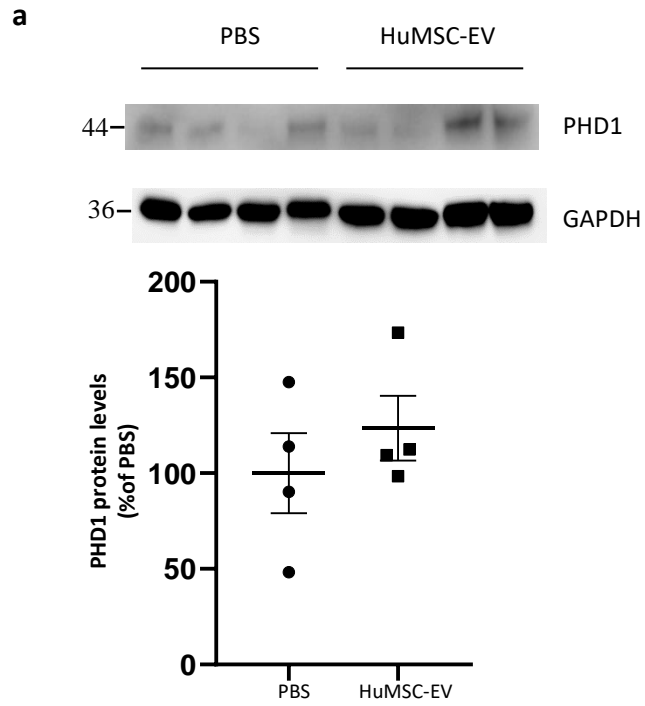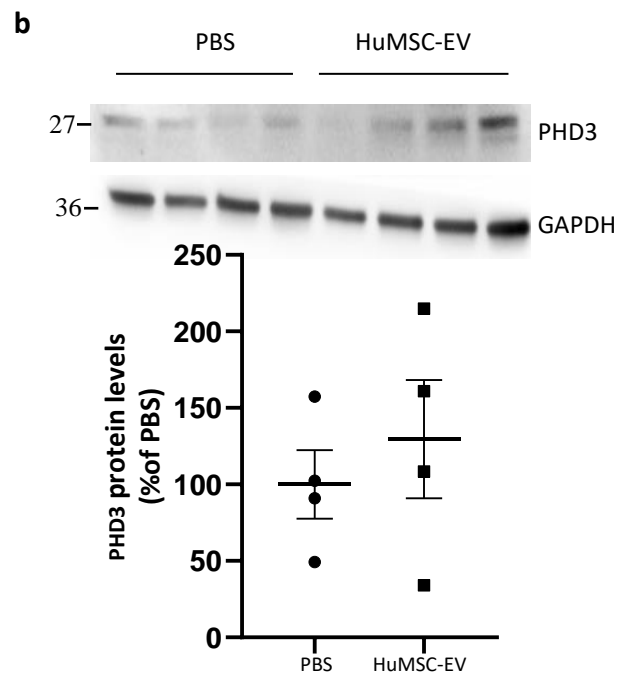

**Figure S1**

Supplement: Supplementary file 3 — Figure S1 [file 41420_2023_1335_MOESM3_ESM.pdf]

## Dermal endothelial cells

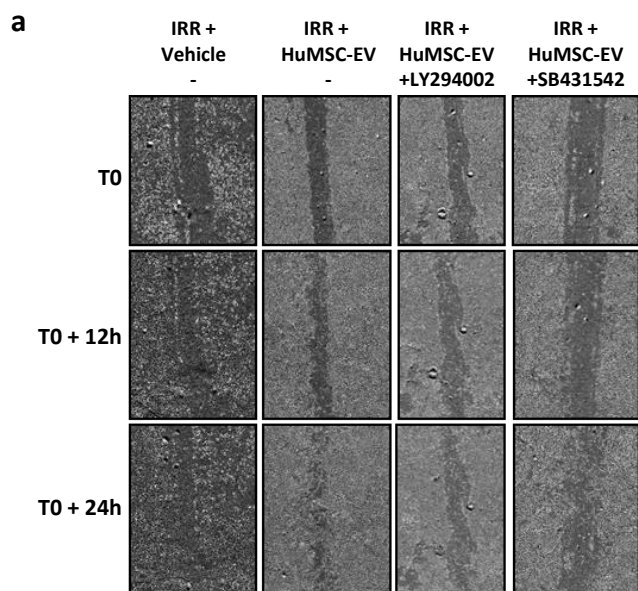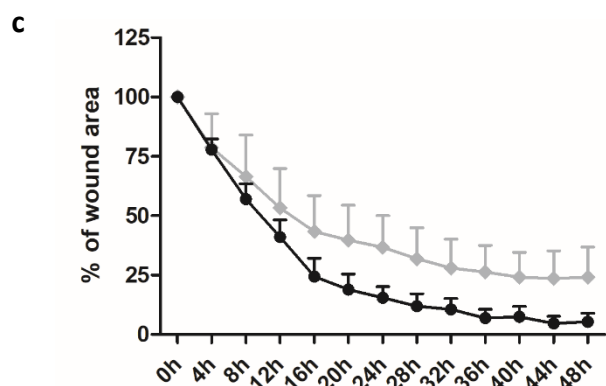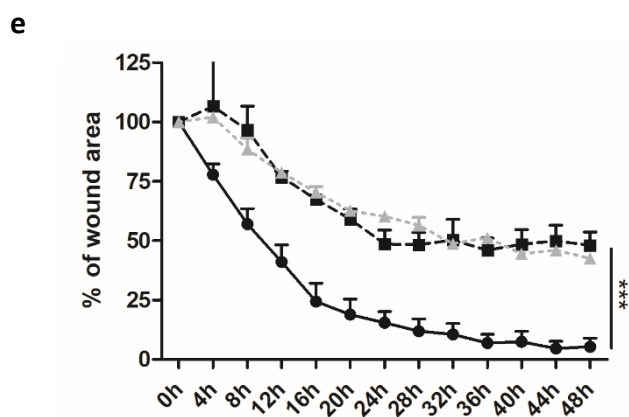

## Fibroblasts

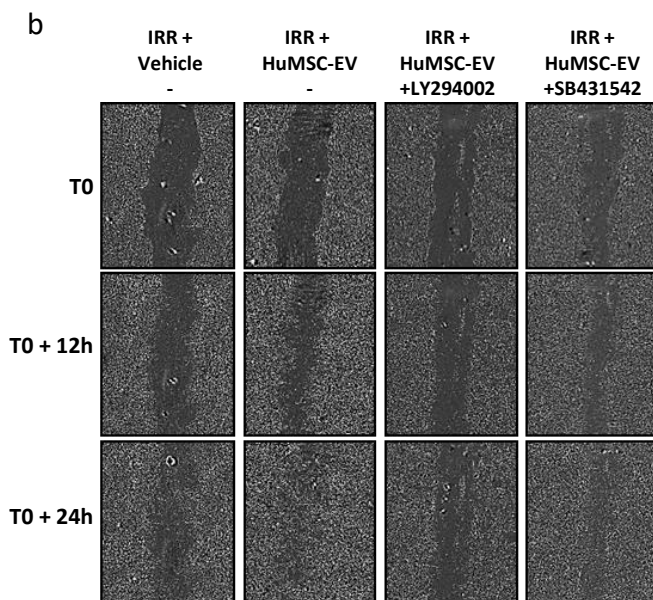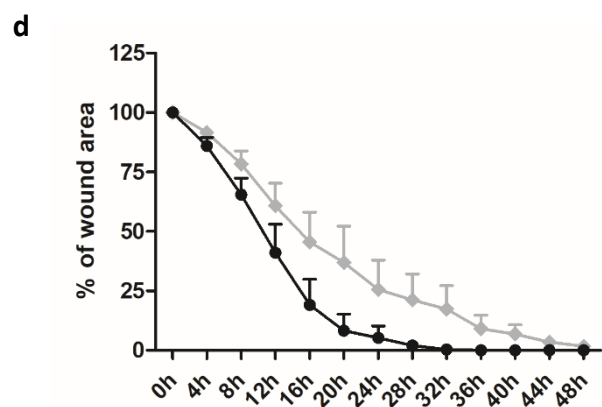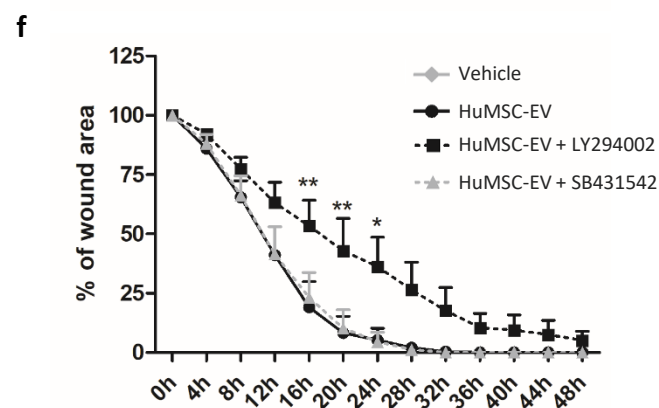

Figure S2

Supplement: Supplementary file 4 — Figure S2 [file 41420_2023_1335_MOESM4_ESM.pdf]
